# Changes of the glutathione redox system during the weaning transition in piglets, in relation to small intestinal morphology and barrier function

**DOI:** 10.1186/s40104-020-00440-7

**Published:** 2020-04-23

**Authors:** Jeroen Degroote, Hans Vergauwen, Wei Wang, Chris Van Ginneken, Stefaan De Smet, Joris Michiels

**Affiliations:** 1 Laboratory for Animal Nutrition and Animal Product Quality (LANUPRO), Department of Animal Sciences and Aquatic Ecology, Faculty of Bioscience Engineering, Ghent University, Block F, Campus Coupure, Coupure Links 653, 9000 Ghent, Belgium; 2 Laboratory of Applied Veterinary Morphology, Department of Veterinary Sciences, Faculty of Biomedical, Pharmaceutical and Veterinary Sciences, University of Antwerp, Universiteitsplein 1, Wilrijk, Belgium

**Keywords:** Barrier function, Glutathione, Oxidative stress, Redox status, Small intestine, Weaned piglet

## Abstract

**Background:**

Weaning is known to result in barrier dysfunction and villus atrophy in the immediate post-weaning phase, and the magnitude of these responses is hypothesized to correlate with changes in the glutathione (GSH) redox system. Therefore, these parameters were simultaneously measured throughout the weaning phase, in piglets differing in birth weight category and weaning age, as these pre-weaning factors are important determinants for the weaning transition. Low birth weight (LBW) and normal birth weight (NBW) littermates were assigned to one of three weaning treatments; i.e. weaning at 3 weeks of age (3w), weaning at 4 weeks of age (4w) and removal from the sow at 3 d of age and fed a milk replacer until weaning at 3 weeks of age (3d3w). For each of these treatments, six LBW and six NBW piglets were euthanized at 0, 2, 5, 12 or 28 d post-weaning piglets, adding up 180 piglets.

**Results:**

Weaning increased the glutathione peroxidase activity on d 5 post-weaning in plasma, and duodenal and jejunal mucosa. Small intestinal glutathione-S-transferase activity gradually increased until d 12 post-weaning, and this was combined with a progressive rise of mucosal GSH up till d 12 post-weaning. Oxidation of the GSH redox status (GSH/GSSG E_h_) was only observed in the small intestinal mucosa of 3d3w weaned piglets at d 5 post-weaning. These piglets also demonstrated increased fluorescein isothiocyanate dextran (FD4) and horseradish peroxidase fluxes in the duodenum and distal jejunum during the experiment, and specifically demonstrated increased FD4 fluxes at d 2 to d 5 post-weaning. On the other hand, profound villus atrophy was observed during the weaning transition for all weaning treatments. Finally, LBW and NBW piglets did not demonstrate notable differences in GSH redox status, small intestinal barrier function and histo-morphology throughout the experiment.

**Conclusion:**

Although moderate changes in the GSH redox system were observed upon weaning, the GSH redox status remained at a steady state level in 3w and 4w weaned piglets and was therefore not associated with weaning induced villus atrophy. Conversely, 3d3w weaned piglets demonstrated GSH redox imbalance in the small intestinal mucosa, and this co-occurred with a temporal malfunction of their intestinal barrier function.

## Background

It is well known that abrupt weaning presents a challenge for the intestinal tract, as it needs to instantaneously adapt to sudden changes in feed intake, diet form and composition, nutrient digestibility and social and physical environment. Altogether, these changes induce stress [1], and potentially also induces inflammation and stimulation of the immune system [2]. As a result, weaning is associated with decreased gut health, most often documented by villus atrophy and impairment of the mucosal barrier function in especially the small intestine [3, 4]. Hence, decreased animal performance, higher mortality and excessive use of antimicrobials are to date a major concern in pig production. Extensive research efforts have been made towards unraveling the mechanisms behind weaning induced alterations of gut health. The impact of age at weaning, gut maturation, the immediate post-weaning feed intake and social stress on digestive physiology, microbial and immunological processes are well documented [5–10] and together provide insight and opportunities for mitigation. Recent developments in redox biology might add to this and could particularly further elaborate on the relationship between the epithelial barrier function and villus atrophy. The concepts of redox biology extend beyond ordinary damage control (reactive oxygen species damage DNA, proteins and lipids and thereby affect cell functionality) in an event of oxidative stress. Importantly, redox biology also involves redox switches, e.g. redox sensitive cysteine residues in proteins, which enable fast post-translational modifications of proteins and thereby impact on their function [11]. These mechanisms offer a comprehensive view on tight junction regulation [12–15] and cell cycling from proliferation to apoptosis [16, 17], and hence link barrier function and cell turnover in the intestinal epithelium. Substantial evidence indicates the pivotal role of redox molecules such as glutathione (GSH). Together with its related enzymes, i.e. glutathione peroxidase (GPx) and glutathione-S-transferase (GST), the GSH redox system offers the opportunity to efficiently buffer undesirable oxidation reactions. When oxidized, GSH is converted to glutathione disulphide (GSSG), which is instantaneously reduced back to GSH by glutathione reductase. Cells tightly regulate these oxidation and reduction reactions and strive to keep GSH in a predominantly reduced status. By the law of Nernst, this status is expressed by its half-cell redox potential (GSH/GSSG E_h_) [18]. As this system is under catalytic control at multiple levels, disturbance of this steady state level is therefore considered to represent a thorough change in the redox environment of a tissue [18]. The fact that GSH not only functions as an indicator or buffer, but also actively partakes in the reversible formation of mixed disulfide through protein-S-glutathionylation, underlines the potential impact of these mechanisms in stressful events [11]. Although very fascinating, these complex insights, mainly generated in *in vitro* models, are not easily translated to *in vivo* research. Recent efforts however started to reveal the extent of weaning induced alterations of the antioxidant and redox system *in vivo* [19–27]. Nevertheless, it remains hard to formulate general patterns for antioxidant and redox biomarkers in relation to weaning. This is due to the vast amount of different biomarkers in use, the likelihood that reactions upon weaning are tissue specific [26], and the fact that numerous dietary and animal related factors influence the magnitude of weaning induced responses. The objective of this study was therefore to determine the responses of the antioxidant system upon weaning, with the focus on the small intestinal GSH redox system, in piglets that were subjected to different pre-weaning conditions. A vast body of literature namely documents how functional [7, 28, 29] and also GSH redox [30–32] characteristics of the small intestine depend on birth weight and weaning age. Several authors report compromised digestive functions in suckling and fully weaned piglets that exhibit intrauterine growth restriction (IUGR) [28]. Some papers link this impaired gut health to alterations in the expression of redox-sensitive genes or proteins [30, 33]. Also weaning age, and particularly early weaning, repeatedly showed to impact on gut physiology [6, 29, 34]. In this respect, an earlier study demonstrated how artificial rearing of piglets, i.e. removal from the sow at 3 d of age and feeding a milk replacer, resulted in GSH redox imbalance and barrier disruption in the small intestine in the pre-weaning phase [31]. In the current study, we therefore aim to describe the post-weaning changes of the GSH redox system and explore the relations with the small intestinal integrity during the entire weaning transition in pigs with a low and normal birth weight that are subjected to different pre-weaning strategies.

## Methods

### Experimental setup

This experiment included 180 piglets (Piétrain × Topigs hybrid) selected during 5 consecutive farrowing rounds from 68 litters with 14 or more live born piglets. At the day of birth, 90 pairs of low birth weight (LBW) and normal birth weight (NBW) gender-matched littermates were selected based on criteria parallel to previous research [30, 31]. Piglets having a birth weight (BiW) between 0.75 and 0.90 kg and belonging to the lower quartile of their litter birth weight were classified as LBW, whereas an NBW littermate had a birth weight within 0.5 SD units of the mean birth weight of the whole litter. Cross-fostered piglets were excluded for the study. Pairs of LBW and NBW littermates were assigned to one of three weaning treatments (WT); i.e. weaning at 3 weeks of age (19.6 ± 0.50 d) (3w), weaning at 4 weeks of age (26.6 ± 0.50 d) (4w) and removal from the sow at 3 d of age and feeding a milk replacer until weaning at 3 weeks of age (19.8 ± 0.38 d) (3d3w). These 3d3w weaned piglets were raised following the procedures described by De Vos et al. [35] and Vergauwen et al. [31], using a commercial brooder system (Rescue Deck®) where milk replacer was mixed with water at a ratio of 1/4 (*w*/*w*), and was provided by an automated milk dispensing system. After weaning, piglets were housed in groups of 6 piglets per pen (2.40 m × 1.25 m). Pens were equipped with a full slatted floor, drinker nipple and feeder trough. Piglets belonging to different birth weight and WT groups were housed in separate pens. All piglets were fed the same weaner diet ad libitum until sampling at either 0, 2, 5, 12 or 28 d post-weaning (DPW), where sampling a d 0 implicates transport to the lab and sampling within 6 h after maternal separation. Body weight was recorded at birth, weaning and sampling. Composition of the milk replacer and weaner diet can be found in annex (Additional file 1: Table S1). The experimental setup resulted in 30 different animal groups (2 × 3 × 5 factorial design; Biw, WT and DPW, respectively) with 6 piglets per group.

### Sample collection

Piglets were sedated by electrical stunning and blood was collected during exsanguination in EDTA and heparin tubes containing supplemental bathophenanthroline disulfonate sodium salt. Subsequently, the gastrointestinal tract was dissected and the small intestine and liver were isolated. The length of the small intestine was measured. Both at 5% (duodenum) and 75% (distal jejunum) of the total length of the small intestine, three tissue samples, measuring 10 cm, 5 cm and 20 cm, were taken, rinsed with a 0.9% NaCl-solution, and used in their respective order for Ussing chamber measurements, histo-morphology, and collection of the mucosa by scraping the mucosal surface with a glass slide. Areas containing Peyer’s patches were avoided.

### Oxidative and redox status

Blood plasma was collected from EDTA tubes after centrifugation (3,000 × g, 15 min), and was stored at − 20 °C until analysis of the oxygen radical absorbance capacity (ORAC), malondialdehyde (MDA) and GPx activity. ORAC was determined by the assay of Ou et al. [36]. The thiobarbituric acid reactive substances method [37] was used to quantify MDA as a marker for lipid peroxidation. GPx activity was determined spectrophotometrically by principles described by Hernandez et al. [38]. Erythrocytes were harvested by centrifuging (3,000 × g, 15 min) 0.5 mL of heparinized blood, and removing the residual plasma. Erythrocytes were lysed with 70% metaphosphoric acid solution and intense vortexing. An aliquot was transferred to a vial, containing γ-glutamyl-glutamate as an internal standard, and was snap frozen in liquid nitrogen before storing at − 80 °C. These samples were analyzed for GSH and GSSG. In addition, duodenal and jejunal tissue samples were slit longitudinally and mucosa was obtained by scraping off the surface with a glass slide. A sample was also taken central in the right lateral lobe of the liver. Mucosal and liver samples were further treated according to the following procedure. A first tissue subsample was instantaneously turned into a phosphate buffered mucosal homogenate using 1% triton-X-100 phosphate buffer (pH 7.0, 50 mmol/L). An aliquot was taken to determine total protein content by the biuret method, before centrifuging the homogenate (13,000 × *g*, 15 min, 4 °C). The supernatants was snap frozen and stored at − 80 °C pending the analysis of GPx, MDA and GST. Here, GST was determined by measuring the increase in absorbance at 340 nm associated with the conjugation of GSH with 1-chloro-2,4-dinitrobenzene catalyzed by GST [39]. A second subsample was instantaneously homogenized in a 10% perchloric acid solution. After centrifugation (13,000 × *g*, 15 min, 4 °C), the resulting acid extract was transferred to a tube containing a γ-glutamyl-glutamate internal standard solution. Samples were then snap frozen in liquid nitrogen and stored at − 80 °C. Later, GSH and GSSG concentrations were quantitated by high-performance liquid chromatography analysis [40, 41]. In brief, this method involves iodoacetic acid as a thiol quenching agent, 1-chloro-2,4-dinitrobenzene as derivatization reagent, reversed-phase HPLC separation on an aminopropyl column, and absorption measurement at 365 nm. The GSH and GSSG concentrations were determined relative to internal and external standard solutions and were expressed on protein content. The GSH/GSSG E_h_ value was calculated by using the appropriate forms of the Nernst equation, assuming a pH of 7.4 and a temperature of 37 °C [18].

GSH/GSSG E_h_(mV) =  − 264 – 61.5/2 × log_10_(GSH^2^/GSSG).

### Histo-morphology

Small intestinal samples were placed in 4% phosphate buffered formalin (pH 7.4) for 2 h at room temperature. After rinsing with phosphate buffered saline, the tissue was dehydrated and paraffin embedded according to standard procedures. Transverse 5 μm sections were stained with hematoxylin and eosin. Villus length, mid-villus width and crypt depth were assessed in 30 well oriented villi and adjacent crypts using image analysis software (Olympus BX 61, analySIS Pros, Aartselaar, Belgium).

### Ussing chamber macromolecular permeability

The apparent permeability coefficients (P_app_) of two macro-molecular markers; fluorescein isothiocyanate dextran 4 kDa (FD4) and horseradish peroxidase 40 kDa (HRP) was determined following experimental procedures described by Neirinckx et al. [42] and Michiels et al. [28]. Duodenal and jejunal segments were placed in oxygenated (O_2_/CO_2_ 95/5) Ringer’s buffer (pH 7.4, 115 mmol/L NaCl, 5 mmol/L KCl, 25 mmol/L NaHCO_3_, 2.4 mmol/L Na_2_HPO_4_, 1.25 mmol/L CaCl_2_, 1 mmol/L MgSO_4_) solution at 38 °C until processing and mounting within 10 min post-mortem. The mucosal and submucosal layer were stripped of its serosal and muscle layers, and the segments were opened longitudinally along the mesenteric border. At each intestinal site, two pieces of stripped tissue per piglet were mounted in Ussing chambers (Dipl.-ing Muβler Scientific Instruments, Aachen, Germany) with an exposed surface area of 1.07 cm^2^. Areas containing Peyer’s patches were not exposed to the open surface area. Both mucosal and serosal compartments were simultaneously filled with 6.5 mL warm (38 °C) Ringer’s buffer containing 6 mmol/L mannitol and glucose, respectively. The system was water-jacketed to 37 °C and the buffer compartments were oxygenated with a O_2_/CO_2_ (95/5) gas flow. After an equilibration period of 20 min, FD4 (4 kDa, Sigma-Aldrich, Bornem, Belgium) and HRP (40 kDa, type IV, Sigma-Aldrich, Bornem, Belgium) were added to the buffer compartment at the mucosal side to reach a final concentration of 0.8 mg/mL FD4 and 0.4 mg/mL HRP. 200 μL Samples were taken at the serosal buffer compartment at 20, 40, 60 and 80 min after adding the markers, whilst the same volume of buffer solution was taken from the mucosal buffer compartment to maintain volume balance across sides. Buffer compartments were wrapped in aluminum foil to provide light protection. 200 μL Samples were stored at − 20 °C in light resistant amber polypropylene copolymer vials until analysis. Fluorescence intensity of FD4 was measured at λ_exc_ of 485 nm and λ_em_ of 538 nm using a fluorescence plate reader (Thermo Scientific, Marietta, Ohio, USA). The HRP activity was measured by a modification of the Worthington method [43], following the rate of increase in optical density at 460 nm using a microplate absorbance reader (IMark, Bio-Rad, Hercules, California, USA). As in previous research [30, 31, 44], the change in concentration of marker solution (dc/dt) in the acceptor compartment between 20 and 100 min, the buffer volume in the donor compartment (V), the initial marker concentration in the donor compartment (C_0_), and the exposed tissue surface area (A) were used to calculate the P_app_.

P_app_(cm/s) = dc/dt × V/(A × C_0_).

### Small intestinal protein expression

Similar to Vergauwen et al. [31], commercially available enzyme-linked immunosorbent assays (ELISA) of occludin (SEC228Hu), claudin-3 (SEF293Hu), proliferating cell nuclear antigen (PCNA) (SEA591Hu) and caspase-3 (SEA626Hu) (Cloud-Clone Corporation®, Houston, TX, USA) were used to evaluate the protein concentration of specific tight junction proteins and markers for apoptosis and mitosis. In brief, mucosa scrapings that had been snap frozen in liquid nitrogen and stored at − 80 °C were crushed in liquid nitrogen and lysed in a phosphate-buffered saline solution (pH 7.4, 0.01 mol/L) by sonication. Subsequently, the samples were centrifuged (10,000 × *g*, 2 min, 4 °C) and the supernatant was collected. Next, the total protein concentration was determined using a Pierce™ bicinchoninic acid Assay (BCA) Kit (Thermo Scientific, Rockford, USA), and the supernatant was diluted to protein concentration of 10 ng/μL. Subsequently samples were processed following the manufacturer’s guidelines. Absorbance was measured at 450 nm at 25 °C, and values of protein abundance were expressed as fmol per mg protein.

### Statistical analysis

Data were analyzed using a general linear model procedure (IBM SPSS Statistics version 22). In all analyses, piglet served as the experimental unit. The model included the fixed effects of the three main factors, 1) birth weight (Biw), comparing LBW and NBW piglets, 2) weaning treatment (WT), comparing 3d3w, 3w and 4w weaned piglets and, 3) days post-weaning (DPW) comparing piglets of 0, 2, 5, 12 and 28 d post-weaning, and their two-way interaction terms. A *post-hoc* Tukey test was used to discriminate treatment differences for overall effects of the main study factors. If interaction terms reached *P* ≤ 0.10, a second analysis was done where the effect of the first factor of the interaction term was tested for each experimental group of the second factor separately, and vice versa. Date are expressed as least squares means with their standard error (SE) in figures, or alternatively with the standard error of the grand mean (SEM) in tables. Differences were declared significant at *P* ≤ 0.05.

## Results

### Animal performance

Several significant treatment effects and interactions on pre- and post-weaning performance were observed (Table 1). Selection on birth weight resulted in an LBW and an NBW group having a mean birth weight of respectively 0.85 ± 0.09 kg and 1.37 ± 18 kg (mean ± SD; *P* < 0.001). Pre-weaning average daily gain (ADG) was reduced by 29% in LBW piglets, resulting in a 31% lower average weaning weight. Besides Biw, also WT affected weaning weight. Due to the significant higher pre-weaning ADG (+ 89 g/d) in 3d3w weaned piglets, this group exhibited a significantly higher weaning weight than piglets weaned at 3w (+ 1.77 kg) and 4w of age (+ 0.43 kg). Weaning weight was significantly higher in piglets weaned at 4w versus those weaned at 3w of age. This was however due to the difference in age, as pre-weaning ADG was not different between 3w and 4w weaned piglets. A significant interaction for WT × DPW was observed regarding the weaning weight (*P* = 0.033). Apparently, *at random* allocation did not result in a perfect representation of the overall effect of WT for piglets sampled on different days post-weaning. Nevertheless, stratification for weaning weight across the different days post-weaning was successful within a weaning treatment (Additional file 2: Table S3), and no interaction terms reached significance regarding the pre-weaning ADG (Table 1).

**Table 1:** Body weights and growth performance of the piglets pre- and post-weaning

	Animals, *n*	Birth weight, kg	Weaning weight, kg	Final weight, kg	ADG pre-weaning, g/d	ADG post-weaning, g/d	ADFI post-weaning, g/d	FCR post-weaning (ADG/ ADFI)
Birth weight category (Biw)								
LBW	90	0.85	4.95	6.64	189	30	145	0.21
NBW	90	1.37	7.14	9.27	266	43	183	0.23
Weaning treatment (WT)								
3d3w	60	1.10	6.78^c^	8.68^b^	287^b^	-6^a^	182^b^	−0.03
3w	60	1.12	5.01^a^	6.70^a^	198^a^	40^b^	140^a^	0.29
4w	60	1.11	6.35^b^	8.49^b^	197^a^	75^c^	168^b^	0.45
Day post-weaning (DPW)								
d 0	36	1.09	5.98	5.98^x^	227	–	–	–
d 2	36	1.09	5.88	5.47^x^	223	-203^w^	44^w^	−4.61^y^
d 5	36	1.14	6.20	6.01^x^	233	-37^x^	107^x^	−0.35^yz^
d 12	36	1.16	6.13	7.46^y^	230	113^y^	222^y^	0.51^yz^
d 28	36	1.08	6.03	14.86^z^	226	309^z^	444^z^	0.70^z^
SEM	–	0.01	0.07	0.10	3	5	3	0.34
*P*-value								
BiW		< 0.001	< 0.001	< 0.001	< 0.001	0.163		
WT		0.842	< 0.001	< 0.001	< 0.001	< 0.001		
DPW		0.089	0.634	< 0.001	0.876	< 0.001		
BiW×DPW		0.321	0.990	0.012	0.960	0.005		
WT×DPW		0.472	0.033	0.037	0.074	< 0.001		
BiW×WT		0.989	0.864	0.863	0.101	0.560		

Birth weight (BiW), weaning treatment (WT), days post-weaning (DPW), Average daily gain (ADG), average daily feed intake (ADFI), feed conversion ratio (FCR). Piglets were either weaned at 3 weeks of age (3w), 4 weeks of age (4w), or separated from the sow at 3d of age and fed a milk replacer until weaning at 3w of age (3d3w), and either had a low birth weight or normal birth weight (NBW). ^a,b,c^ Within column, means without a common superscript differ for the effect of weaning treatment (WT: *P* ≤ 0.05). ^w,x,y,z^ Within column, means without a common superscript differ for the effect of days post-weaning (DPW: *P* ≤ 0.05).

After weaning, Biw, WT and DPW all significantly influenced body weight at sampling. On average, significant body weight loss was observed at d 2 post-weaning, and piglets caught up with their initial weight on d 5 post-weaning (DPW: *P* < 0.001). This post-weaning growth check was however larger in 3d3w weaned piglets, as 3d3w weaned piglets exhibited lower ADG (− 379 g/d) compared to 3w (− 134 g/d) and 4w (− 96 g/d) weaned piglets (WT × DPW: *P* < 0.001) on d2 post-weaning. A similar effect was seen on d 5 post-weaning, where ADG was still significantly lower in 3d3w versus 4w weaned piglets. Nevertheless, 3d3w weaned piglets reached a significantly higher body weight at d 28 post-weaning compared to 3w and 4w weaned piglets (Additional file 2: Table S4). Next, although Biw did not significantly impact post-weaning ADG (*P* = 0.163), the effect of Biw was found to depend on the day post-weaning (BW × DPW: *P* = 0.005). LBW piglets sampled at 2 or 5 d post-weaning did not differ in ADG from NBW piglets (*P* < 0.05) (Additional file 2: Table S3) but they started to have a reduced ADG when kept until 12 or 28 d post-weaning (*P* ≤ 0.05).

### The oxidative status and glutathione redox system

The total antioxidant capacity of plasma was assessed by means of the ORAC assay (Table 2). The ORAC values significantly dropped from d 2 to d 12 and d 28 post-weaning (DPW: *P* < 0.001) and were lower in 3d3w weaned piglets compared to 4w weaned piglets (*P* < 0.001). Concentrations of MDA (Table 2), a marker for lipid peroxidation, were affected by DPW (*P* < 0.001). Plasma levels were lower on d 5 and d 12 compared with d 0 and d 28 post-weaning. In the duodenal mucosa and liver tissue, MDA concentrations were significantly increased on d 28 versus d 0 post-weaning, opposite to what was found in the distal jejunal mucosa where values were higher on d 0 and d 2 versus d 5, d 12 and d 28 post-weaning (DPW: *P* < 0.001). Distal jejunal MDA levels were also found to be significantly higher in 4w versus 3d3w weaned piglets (WT: *P* = 0.016). Nevertheless, when comparing different weaning treatments, moderate variations in the overall effect of DPW on MDA levels were observed (WT × DPW: *P* < 0.05).

**Table 2:** Effect of birth weight (*n* = 90), weaning treatment (*n* = 60) and days post-weaning (*n* = 36), and their interaction (*n* = 12-30), on oxidative stress biomarkers in weaned piglets

	Birth weight (Biw)		Weaning treatment (WT)			Days post-weaning (DPW)					SEM	*P*-value					
	LBW	NBW	3d3w	3w	4w	d0	d2	d5	d12	d28		BiW	WT	DPW	BiW× DPW	WT× DPW	BiW× WT
ORAC, μmol trolox eq./mL																	
PLA	23.4	23.5	22.6^a^	24.4^b^	23.3^ab^	24.1^y^	24.6^z^	24.2^yz^	22.7^xy^	21.7^x^	0.2	0.901	<0.001	<0.001	0.536	0.252	0.640
MDA, nmol/mg prot.																	
PLA*	10.9	10.7	11.1	10.9	10.5	11.6^z^	11.0^yz^	10.2^y^	10.0^y^	11.3^z^	0.1	0.386	0.127	<0.001	0.713	0.661	0.306
DUO	299	285	288	304	284	261^y^	283^y^	287^yz^	296^yz^	332^z^	6	0.210	0.337	<0.001	0.972	0.040	0.782
JEJ	334	351	312^a^	371^b^	343^ab^	408^z^	416^z^	282^y^	296^y^	310^y^	8	0.301	0.016	<0.001	0.788	<0.001	0.402
LIV	634	650	648	640	637	646^y^	504^x^	632^y^	675^yz^	754^z^	12	0.502	0.926	<0.001	0.825	0.005	0.343

Birth weight (Biw), weaning treatment (WT), day post-weaning (DPW), oxygen radical scavenging activity (ORAC), malondialdehyde (MDA), blood plasma (PLA), duodenal mucosa (DUO), distal jejunal mucosa (JEJ), liver tissue (LIV). Piglets were either weaned at three weeks of age (3w), four weeks of age (4w), or separated from the sow at 3d of age and fed a milk replacer until weaning at 3w of age (3d3w), and either had a low birth weight or normal birth weight (NBW). ^a,b^ Within a row, means without a common superscript differ for the effect of weaning treatment (WT: *P* ≤ 0.05). ^x,y,z^ Within a row, means without a common superscript differ for the effect of days post-weaning (DPW: *P* ≤ 0.05). ^*^ Expressed in nmol/mL plasma

**Fig. 1:**
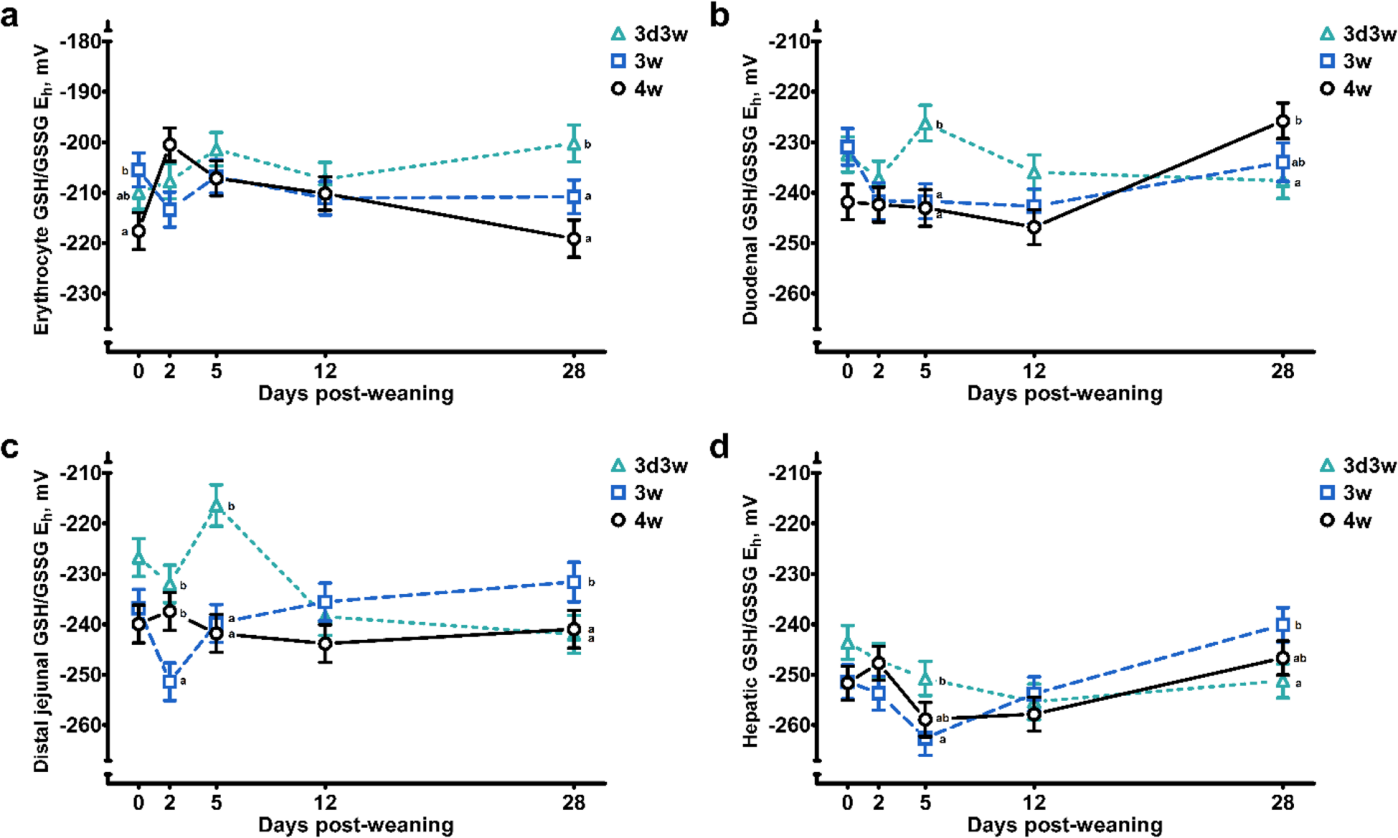
The glutathione redox status (GSH/GSSG E_h_) in (**a**) erythrocytes, (**b**) duodenal mucosa, (**c**) distal jejunal mucosa and (**d**) liver tissue of piglets either weaned at three weeks of age (3w), four weeks of age (4w), or separated from the sow at 3 d of age and fed a milk replacer until weaning at 3w of age (3d3w). Values are least squares means ± SE (*n* = 12 as low birth weight and normal birth weight piglets were pooled together). ^a,b^ Per day post-weaning, means without a common superscript differ for the effect of weaning treatment (WT: *P* ≤ 0.05)

During the weaning transition, GSH levels were affected in erythrocytes (*P* < 0.001), the duodenal mucosa (*P* < 0.001) and the distal jejunal mucosa (*P* = 0.053). On average, GSH concentrations were found to increase significantly towards the end of the weaning transition (d 12 or d 28 post-weaning). Nevertheless, this general pattern was influenced by weaning treatment in erythrocyte and jejunal mucosal (WT × DPN: *P* ≤ 0.05). In erythrocytes, the GSH increase was not observed in 3d3w weaned piglets. Consequently, erythrocyte GSH levels at d 12 and d 28 post-weaning were significantly lower in 3d3w weaned piglets when comparing with 3w and 4w weaned piglets. In the distal jejunum, GSH levels were significantly lower in 3d3w weaned piglets at d 2 and d 5 post-weaning, compared to the age-matched 3w weaned piglets (Additional file 2: Table S4). These differences are also reflected in the average GSH level per weaning treatment, where 3d3w weaned piglets showed the lowest GSH levels in erythrocytes, the duodenal and distal jejunal mucosa (WT: *P* ≤ 0.05). Remarkably, average GSSG levels were increased in the small intestinal mucosa of 3d3w weaned piglets (WT: *P* = 0.05) and GSSG levels were significantly influenced by DPW (*P* ≤ 0.05), as observed for GSH. In general, GSSG levels were highest at d 28 post-weaning in the duodenal mucosa, but in the distal jejunal mucosa, GSSG levels were highest at d 5 post-weaning. Yet again, the effect of DPW on mucosal GSSG levels was influenced by the weaning treatment (WT × DPW: *P* < 0.001). Most importantly, GSSG concentrations in the small intestinal mucosa were increased on d 5 post-weaning in 3d3w weaned piglets, when compared to 3w and 4w weaned piglets (*P* ≤ 0.001) (Additional file 2: Table S4). To evaluate the changes in GSSG relative to the changes in GSH levels, the GSH/GSSG E_h_ gives a comprehensive view of the redox status of the GSH pool. Here, a higher value, i.e. closer to zero, indicates a more oxidized redox status, which can be attributed to increased GSSG and/or reduced GSH levels. Results indicate that GSH/GSSG E_h_ values remain relatively stable in erythrocytes and the distal jejunal mucosa during the weaning transition (DPW: *P* > 0.05). DPW effects were only found in the duodenal mucosa and liver, where a significantly more oxidized GSH pool was observed at d 28 versus d 12 post-weaning. Besides, hepatic GSH redox status was significantly lower at d 5 post-weaning (Additional file 2: Table S2). Importantly, these general patterns were found to differ between different weaning treatments (WT × DPW: *P* ≤ 0.05). These differences are depicted in Fig. 1 and can be found in Table S4 (Additional file 2). It was remarkable that both in the duodenal and distal jejunal mucosa, the GSH/GSSG E_h_ of 3d3w weaned piglets indicated significantly more oxidation on d 5 post-weaning when compared with weaning at 3w and 4w of age. In contrast, the GSH/GSSG E_h_ was in a more reduced state in the distal jejunal mucosa at d 2 post-weaning in 3w weaned piglets, compared to 3d3w and 4w weaned piglets. Secondly, the increase in GSH/GSSG E_h_ at d28 that was observed in duodenal and liver tissue was only seen in 4w and 3w weaned piglets. Next, with regard to erythrocytes, 4w weaned piglets were characterized by a more reduced GSH redox pool on d 0 and d 28 post-weaning. Finally, in accordance with what was observed for GSH and GSSG, the GSH/GSSG E_h_ indicates a more oxidized GSH redox pool in erythrocytes and small intestinal mucosa in 3d3w versus 4w weaned piglets, irrespective of the day post-weaning (WT: *P* < 0.05) (Additional file 2: Table S2).

Concerning the GSH related enzymes, the weaning transition was found to be the most determining factor, as DPW significantly influenced GPx and GST activities in all measured tissues (Table 3). GPx activity peaked on d 5 post-weaning in plasma and small intestinal mucosa. In liver tissue however, this pattern was different, as GPx activities temporarily declined on d 2 post-weaning. Small but significant effects were also found for WT and the interaction term WT × DPW, where 3d3w weaned piglets were characterized by a higher hepatic GPx activity, particularly on d 12 post-weaning, comparing with 4w weaned piglets (Additional file 2: Tables S2 and Table S4). The GST activity was found to peak on d 12 in the distal jejunum. In the duodenum, values were high at d 12 and d 28 post weaning, whereas in the liver, the activity was highest on d 28 post-weaning. Hepatic GST activity was lower in 3w versus in 4w weaned piglets (WT: *P* = 0.010), and this decrease was also observed for these piglets in the small intestinal mucosa at specific days post-weaning (Additional file 2: Table S4) (WT × DPW: *P* < 0.05). Finally, a significant effect of Biw on the GSH redox system was observed. Here, the Biw × WT term indicated a significant effect for the duodenal GST activity. Still, *post-hoc* analysis could not further differentiate treatment groups here. Hence no noteworthy observations can be reported. This leads to the conclusion that DPW and WT had a high impact on the GSH redox system, and no evidence was found differentiating LBW and NBW piglets.

**Table 3:** Effect of birth weight (*n* = 90), weaning treatment (*n* = 60) and days post-weaning (*n* = 36), and their interaction (*n* = 12-30), on the glutathione redox system in weaned piglets

	GSH, μmol/mg prot.				GSSG, μmol/mg prot.				GPx, U/g prot.				GST, U/g prot.		
	ERY^§^	DUO	JEJ	LIV	ERY^§^	DUO	JEJ	LIV	PLA^†^	DUO	JEJ	LIV	DUO	JEJ	LIV
Birth weight category (BiW)															
LBW	955	22	15.6	46.9	60	0.71	0.35	0.85	0.286	8.9	8.6	31.8	28.1	10.9	78
NBW	989	21.9	16.1	43.6	65	0.72	0.38	0.8	0.292	8.7	8.7	31.8	29.6	10.4	76.6
Weaning treatment (WT)															
3d3w	893^a^	20.6^a^	14.5^a^	40.6	69	0.83^b^	0.44^b^	0.8	0.279	8.2^a^	9.5	33.7^b^	28.7	10.4	73.6^ab^
3w	1010^b^	22.1^ab^	16.9^b^	44	62	0.64^a^	0.36^ab^	0.78	0.285	9.6^b^	8.1	31.4^ab^	27.9	10.8	69.7^a^
4w	1012^b^	23.2^b^	16.2^ab^	51.2	56	0.68^ab^	0.30^a^	0.9	0.302	8.6^ab^	8.4	30.3^a^	29.9	10.7	88.6^b^
Day post-weaning (DPW)															
d 0	938^y^	17.8^x^	14.4^y^	51.3	56	0.55^xy^	0.35^yz^	0.89^yz^	0.235^x^	8.2^yz^	7.1^y^	33.4^z^	22.3^y^	10.7^yz^	81.5^yz^
d 2	881^y^	17.6^x^	15.8^yz^	39.9	57	0.38^x^	0.26^y^	0.73^y^	0.280^xy^	9.9^z^	9.7^z^	28.0^y^	20.5^y^	10.2^yz^	56.0^x^
d 5	842^y^	22.1^y^	16.3^yz^	44.4	67	0.74^y^	0.48^z^	0.61^y^	0.373^z^	9.9^z^	10.6^z^	32.1^yz^	26.5^y^	8.2^y^	67.9^xy^
d 12	997^y^	27.3^z^	18.1^z^	50.1	60	0.75^y^	0.41^yz^	0.73^y^	0.304^y^	8.7^yz^	9.8^z^	33.2^z^	37.1^z^	12.5^z^	78.4^xy^
d 28	1201^z^	25.0^xy^	14.0^yz^	40.7	73	1.17^z^	0.32^yz^	1.17^z^	0.253^x^	7.3^y^	6.3^y^	32.4^yz^	37.9^z^	11.6^yz^	102.8^z^
SEM	21	0.4	0.4	2.4	2	0.03	0.02	0.05	0.005	0.2	0.3	0.5	1.1	0.4	2.7
*P*-value															
BiW	0.416	0.850	0.616	0.499	0.353	0.882	0.502	0.565	0.615	0.758	0.892	0.998	0.474	0.544	0.798
WT	0.033	0.027	0.054	0.195	0.072	0.051	0.054	0.478	0.195	0.018	0.121	0.032	0.736	0.939	0.010
DPW	< 0.001	< 0.001	0.053	0.450	0.085	< 0.001	0.029	0.002	< 0.001	< 0.001	< 0.001	0.009	< 0.001	0.013	< 0.001
BiW×DPW	0.687	0.682	0.416	0.211	0.983	0.200	0.796	0.891	0.807	0.525	0.344	0.645	0.475	0.580	0.887
WT×DPW	< 0.001	0.067	0.026	0.276	0.143	< 0.001	< 0.001	0.001	0.040	0.325	0.738	0.049	0.039	0.012	0.874
BiW×WT	0.493	0.123	0.391	0.654	0.906	0.423	0.999	0.966	0.676	0.523	0.896	0.310	0.043	0.763	0.930

Birth weight (BiW), weaning treatment (WT), days post-weaning (DPW), glutathione (GSH), glutathione disulphide (GSSG), glutathione peroxidase (GPx), glutathione transferase (GST), duodenal mucosa (DUO), distal jejunal mucosa (JEJ), liver tissue (LIV). Piglets were either weaned at 3 weeks of age (3w), 4 weeks of age (4w), or separated from the sow at 3 d of age and fed a milk replacer until weaning at 3w of age (3d3w), and either had a low birth weight or normal birth weight (NBW). Values are least squares means. ^a,b^ Within a column, means without a common superscript differ for the effect of weaning treatment (WT: *P* ≤ 0.05). ^x,y,z^ Within a column, means without a common superscript differ for the effect of days post-weaning (DPW: *P* ≤ 0.05). ^§^ Expressed in nmol/mL blood. † Expressed in U/mL blood plasma

### Small intestinal histo-morphology and mitotic/apoptotic protein expression

**Fig. 2:**
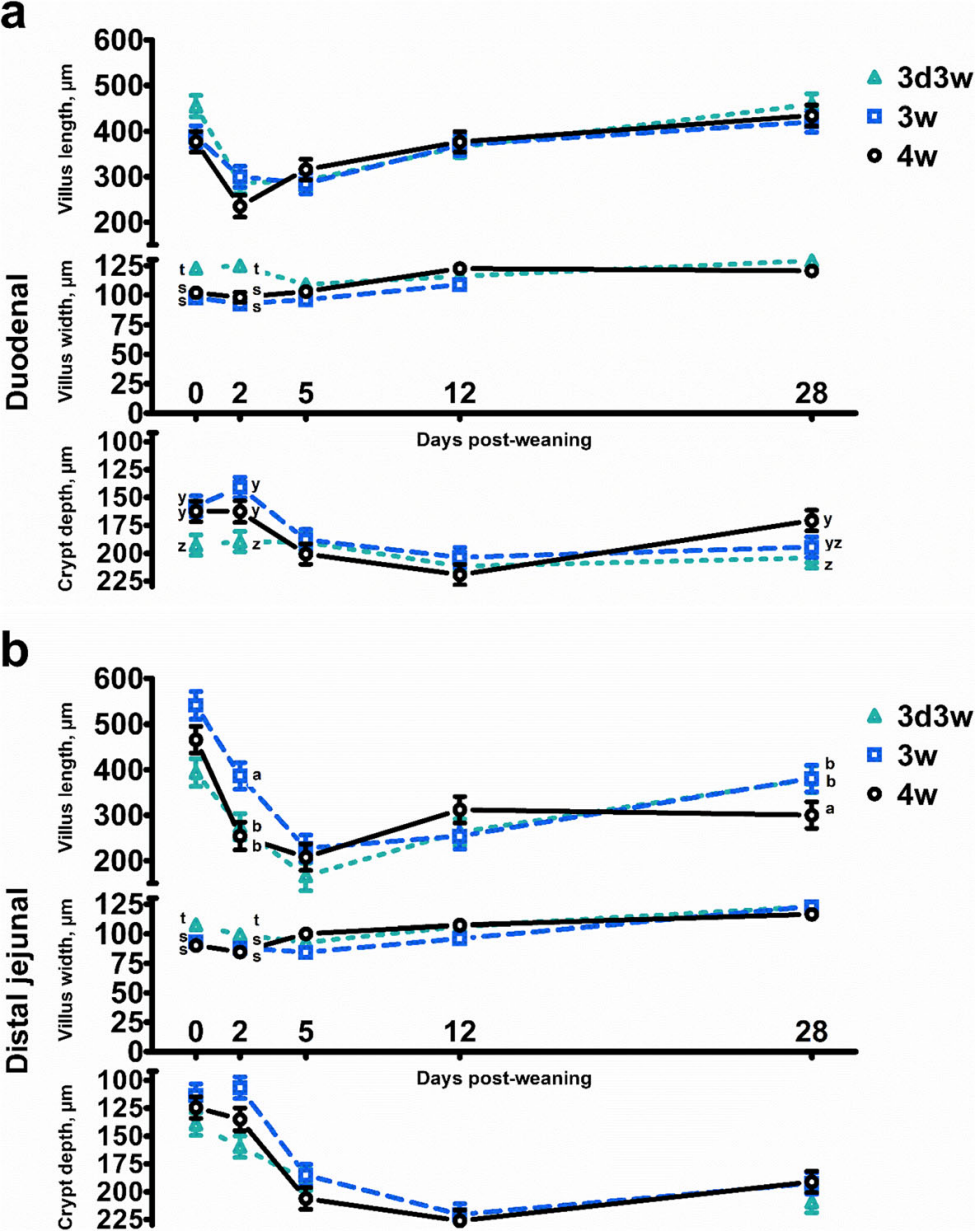
Villus length, villus width and crypt depth (**a**) in the duodenum and (**b**) distal jejunum of piglets either weaned at three weeks of age (3w), four weeks of age (4w), or separated from the sow at 3 d of age and fed a milk replacer until weaning at 3w of age (3d3w). Values are least squares means ± SE (*n* = 12 as low birth weight and normal birth weight piglets were pooled together). ^a,b^ Per day post-weaning, means for villus length without a common superscript differ for the effect of weaning treatment (WT: *P* ≤ 0.05). ^s,t^ Per day post-weaning, means for villus width without a common superscript differ for the effect of weaning treatment (WT: *P* ≤ 0.05). ^y,z^ Per day post-weaning, means for crypt depth without a common superscript differ for the effect of weaning treatment (WT: *P* ≤ 0.05)

Figure 2 illustrates the most important treatment effects on the duodenal and distal jejunal histo-morphology. Overall findings are available in Table S2 (Additional file 2). Generally, weaning was followed by shortening of the villi in the duodenum on d 2 post-weaning, and gradual recovery up to the original length and widening of the villi on d 28 post-weaning. Crypt depth also increased upon weaning (DPW: *P*<0.001), and crypts reached their maximum depth on d 12 post-weaning. These patterns were however somewhat different for 3d3w weaned piglets (Additional file 2: Table S4). Villus width and crypt depth were significantly higher in the duodenum on d 0 and d 2 post-weaning, when compared to 3w and 4w weaned piglets. In the distal jejunum, post-weaning changes to histo-morphological indices were more outspoken, as villus atrophy persisted until d 5 post-weaning, and villi were still 114 μm shorter on d 28 as compared to d 0 post-weaning. Villus length was also found to be higher in 3w weaned piglets compared to 3d3w and 4w weaned piglets (WT: *P* = 0.002), and this was attributed to significantly higher values on d 2 and d 28 post-weaning (WT × DPW: *P* = 0.010). Widening of the jejunal villi took place up till d 28 post-weaning (DPW: *P* < 0.001), and an increased villi width was found in 3d3w weaned piglets on d 0 and d 2 post-weaning (WT × DPW: *P* = 0.007). Distal jejunal crypts reached their maximum size at d 12 post-weaning and were on average deeper in 3d3w compared to 3w weaned piglets (WT: *P* = 0.001). The factor Biw did not significantly affect these three histo-morphology parameters and did not interact with WT or DPW.

**Fig. 3:**
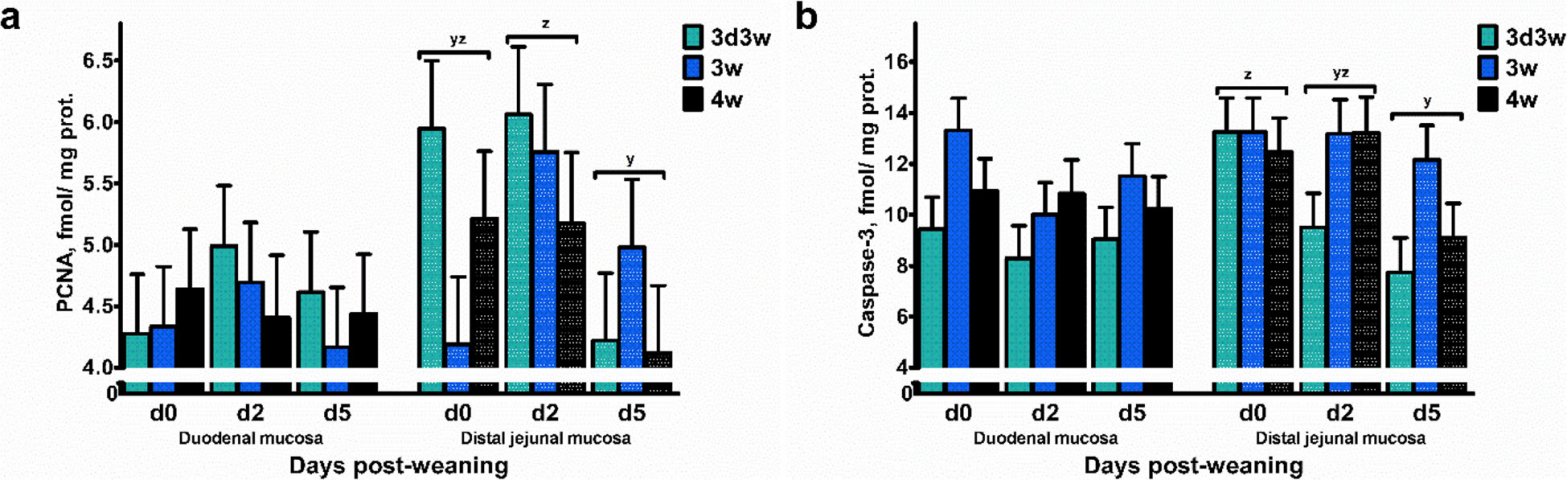
Protein expression of (**a**) proliferating cell nuclear antigen (PCNA) and (**b**) caspase-3 in the duodenal and distal jejunal mucosa of piglets either weaned at 3 weeks of age (3w), 4 weeks of age (4w), or separated from the sow at 3 d of age and fed a milk replacer until weaning at 3w of age (3d3w). Values are least squares means + SE (*n* = 12 as low birth weight and normal birth weight piglets were pooled together). ^y,z^ Means without a common superscript differ for the effect of days post-weaning (DPW: *P* ≤ 0.05)

Concerning the PCNA and caspase-3 protein expression (Fig. 3), effects were limited to the main study factors (Additional file 2: Table S2). The PCNA expression was affected in the distal jejunal mucosa, where values were lower on d 5 versus d 2 post-weaning (DPW: *P* = 0.029). Caspase-3 was also significanlty reduced on d 5, when compared with d 0 post-weaning (DPW: *P* = 0.010). Average caspase-3 expression was lower in 3d3w weaned piglets, when comparing with 3w weaned piglets (WT: *P* = 0.052). Birth weight did not affect the presence of these proteins in the small intestinal mucosa (*P* > 0.05).

### Small intestinal barrier function and tight junction protein expression

**Fig. 4:**
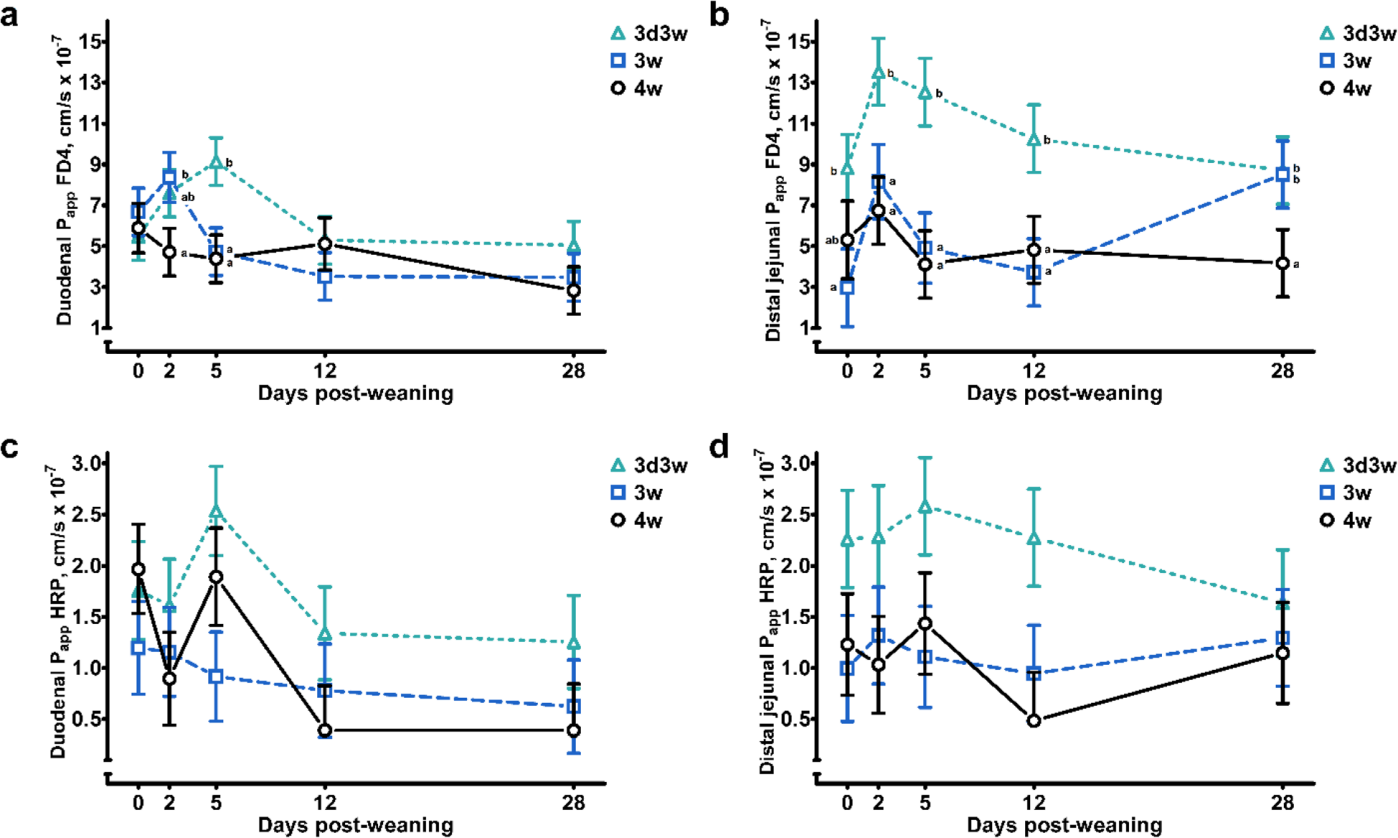
Mucosal apparent permeability coefficient (P_app_) of fluorescein isothiocyanate dextran 4 kDa (FD4) (**a**) at the duodenum and (**b**) distal jejunum, and horseradish peroxidase 40 kDa (HRP) (**c**) at the duodenum and (**d**) distal jejunum of piglets either weaned at 3 weeks of age (3w), 4 weeks of age (4w), or separated from the sow at 3 d of age and fed a milk replacer until weaning at 3w of age (3d3w). Values are least squares means ± SE (*n* = 12 as low birth weight and normal birth weight piglets were pooled together). ^a,b^ Per day post-weaning, means without a common superscript differ for the effect of weaning treatment (WT: *P* ≤ 0.05)

*Ex vivo* macromolecular permeability was determined in the duodenum and distal jejunal mucosa and was found to be significantly affected throughout the weaning transition (DPW) and in some cases by weaning treatment (Additional file 2: Table S2). The FD4 permeability was increased at d 2 versus d 28 post-weaning in the duodenum (DPW: *P* = 0.013), and at d 2 versus d 0 post-weaning in the distal jejunum (DPW: *P* = 0.017). Moreover, average FD4 permeability was higher in 3d3w weaned piglets (WT: *P* ≤ 0.05). Nevertheless, the interaction of WT × DPW tended to influence these patterns (*P* < 0.10) (Fig. 4) (Additional file 2: Table S4). In the duodenum, FD4 flux was significantly higher on d 5 post-weaning in 3d3w versus 3w and 4w weaned piglets. This was in accordance with FD4 fluxes in the distal jejunum, where major increases were found on d 2, d 5 and d 12 post-weaning in 3d3w weaned piglets, when comparing to both 3w and 4w weaned piglets. Permeability was also affected by DPW in 3w weaned piglets, where the FD4 flux on d 2 post-weaning was significantly increased compared to 4w weaned piglets. At both intestinal sites, the FD4 flux was not affected by DPW in 4w weaned piglets. Contrasting to FD4, the HRP fluxes were not significantly altered the first 5 d post-weaning. In the duodenum however, a significant decline was observed on d 12 and d 28 post-weaning, when comparing with d 0 and d 5 post-weaning. Extraordinarily, again 3d3w weaned piglets showed the highest HRP permeability compared to 3w and 4w weaned piglets. No interaction terms reached significance levels, and neither did Biw influence FD4 or HRP permeability.

**Fig. 5:**
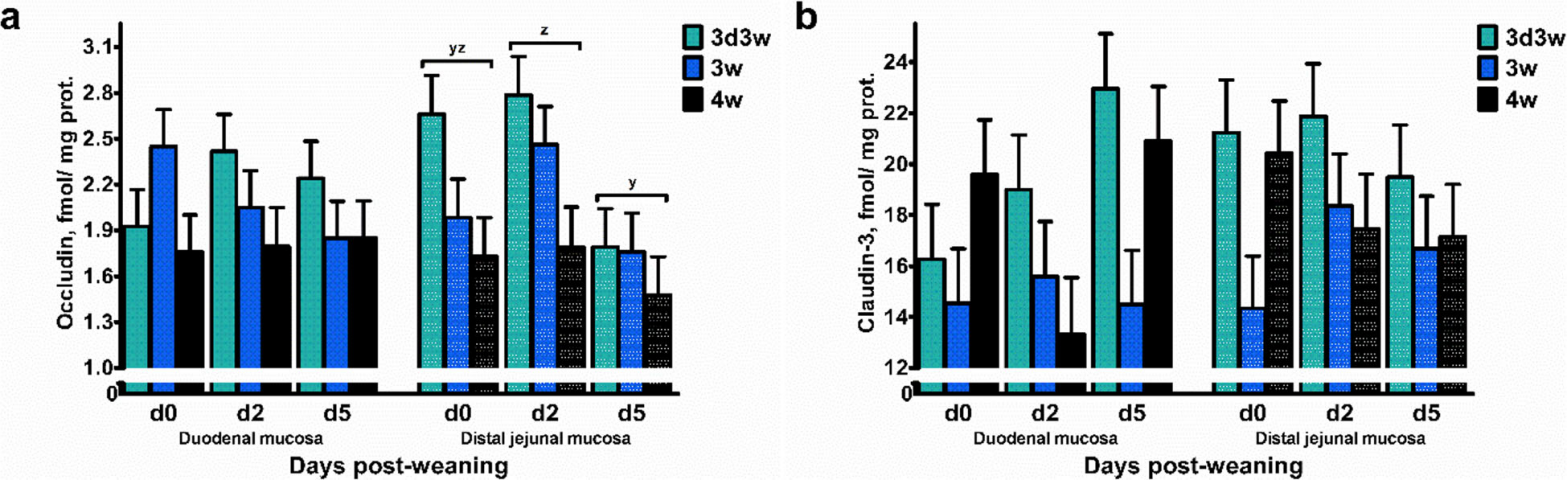
Protein expression of (**a**) occludin and (**b**) claudin-3 in the duodenal and distal jejunal mucosa of piglets either weaned at 3 weeks of age (3w), 4 weeks of age (4w), or separated from the sow at 3 d of age and fed a milk replacer until weaning at 3w of age (3d3w). Values are least squares means ± SE (*n* = 12 as low birth weight and normal birth weight piglets were pooled together). ^y,z^ Means without a common superscript differ for the effect of days post-weaning (DPW: *P* ≤ 0.05)

Figure 5 illustrates the expression of two tight-junction proteins, occludin and claudin-3, in the small intestinal mucosa. Here, DPW only affected the occludin expression in the distal jejunal mucosa, as expression was significantly reduced on d 5 versus d 2 post-weaning (Additional file 2: Table S2). Besides, 3d3w weaned piglets were characterized by a higer expression of occludin in the distal jejunum compared to 4w weaned piglets, and a higher claudin-3 expression at both the duodenum and jejunum compared to 3w weaned pigelts (*P* ≤ 0.05) (Additional file 2: Table S2). Finally, Biw and all interaction terms did not reach significance levels.

## Discussion

### Weaning treatment but not birth weight defines weaning induced alterations of the glutathione redox system

From the results on animal performance, it appears that the experimental setup indeed resulted in groups of piglets that were already quite different at the start of the weaning transition, and in consequence they experienced the weaning transition in a different way. For example, 3d3w weaned piglets experienced markedly more weight loss in the immediate post-weaning period (d 2 and d 5 post-weaning), while on the other hand having a low birth weight resulted in a reduced post-weaning ADG when approaching the end of the weaning transition (d 12 to d 28 post-weaning). Nevertheless, as weaning attenuated performance in all animal groups, it is likely that overall patterns on the oxidative status and GSH redox system can be deduced. And indeed, the weaning transition affected the majority of biomarkers that were analyzed, signaling that changes of the antioxidant system do occur during the weaning transition. Nevertheless, these markers did not coherently point into the direction of severe oxidative stress. For example, MDA changes in the duodenum were reverse to the distal jejunum where a gradual increase and decrease were observed, respectively. A decline in duodenal and distal jejunal MDA was also observed in an earlier study [19]. Even more, hepatic MDA was reduced on d 2 post-weaning. In agreement with Degroote et al. [19, 44], plasma MDA levels were lower on d 5 and d 12 post-weaning. Yet studies comparing weaned piglets with age-matched unweaned controls do report increased MDA levels in serum [23, 45, 46], intestinal mucosa [24, 25] and liver [22] in the first week after weaning. In line with no indication for lipid peroxidation being increased at weaning, the total antioxidant capacity in plasma, assessed by ORAC, was not affected in the immediate post-weaning phase. The antioxidant potential was rather reduced when approaching the end of the weaning transition. These observations seem to correlate to our earlier observations at similar time points [44], whereas other studies reported either unaffected [46, 47] or decreased [22, 24, 25, 45, 48] total antioxidant capacities in the first 2 weeks post-weaning. Yet, interesting evidence is provided by measurements of the GPx activity, as this was clearly increased in plasma and intestinal mucosa on d5-post-weaning. This illustrates a higher requirement for reducing power at that time, and correlates with other studies describing a similar event in plasma [19, 20], small intestinal mucosa [19, 21] and liver [22]. In contrast, hepatic GPx activity was transiently reduced after weaning in the current study, and some authors also report a decreased activity in serum [23] or intestinal mucosa [24, 25] when comparing to age-matched unweaned controls. Next, also the mucosal GST activity gradually increased until d 12 post-weaning, and this was accompanied with a progressive rise of mucosal GSH till d 12 post-weaning, as observed before [19]. As both GSH and GST are required to increase the intestinal conjugation of electrophiles, e.g. lipid hydroperoxides, mycotoxins, etc., their simultaneous increase empowers this function in the small intestine of weaned piglets [49]. It is curious however to not have observed a transient decline in tissue GSH levels upon weaning. This was for example observed in studies of Degroote et al. [26], Li et al., [21] and Robert et al. [20] although admitting these studies only covered two to three time points in the first 2 weeks upon weaning. Altogether, these above-mentioned changes illustrate that the GSH system takes action to prevent oxidative stress and to conserve redox homeostasis, thereby facilitating normal cellular functions [11, 17]. A more complete evaluation of the activity, cellular and subcellular distribution of GSH-related enzymes and other oxidoreductases is however required to assess how these changes facilitate normal cell functions in the gut epithelium [50–53]. In the current study, the distal jejunal GSH redox status was not affected by weaning. In the duodenum, more oxidation was found on d 28 versus d 12 post-weaning. Nevertheless, in most cases the GSH redox status amounted to − 230 to − 240 mV, which corresponds to a fairly reduced steady state redox environment in the mucosa [17, 54]. Nevertheless, one major event of redox disturbance in the small intestine was observed, that was on d 5 post-weaning in 3d3w weaned piglets. Interestingly, this was caused by oxidation of GSH to GSSG, as GSH levels were not lower than on preceding days. This form of redox disturbance was also found by Degroote et al. [19], although others also described weaning induced GSH depletion [20], and both GSH depletion and oxidation [21, 27]. Nevertheless, intestinal GSH levels on d 5 in 3d3w weaned animals were lower when comparing with other weaning treatments. The low levels persisted in these animals throughout the weaning transition. Thus, piglets separated from the sow and fed a milk replacer until weaning exhibited a different GSH redox response upon weaning compared to piglets that remained with the sow. The cause for this can be partially related to the fact that these animals experienced a very abrupt form of weaning, i.e. being weaned from *ad libitum* access to a milk replacer without pre-weaning access to a creep feed. Body weight loss upon weaning was consequently highest in these animals. Maternal separation at a very young age is also known to affect gut functionality and inflict GSH redox imbalance already in the pre-weaning phase [31]. Finally, in sharp contrast to weaning treatment, the factor birth weight did not display major effects in this trial. Therefore, although several studies describe a decreased antioxidant capacity of LBW piglets in the pre-weaning stage [30, 33] or when fully weaned [28, 32], no evidence was found here to support a differential GSH redox response upon weaning.

### Small intestinal villus atrophy during the weaning transition was not related to disturbance of the mucosal glutathione redox status

As expected, weaning was followed by villus atrophy, widening of the villi and deepening of the crypts in the small intestine [29, 55]. It is widely accepted that these developments represent increased cellular proliferation in the crypt, and apoptosis and sloughing at the villus tip [56–58]. In the current study however, mitotic and apoptotic indices, assessed by the protein expression of PCNA and caspase-3, respectively, did not illustrate this phenomenon. For example, the protein expression of PCNA and caspase-3 were not altered in duodenum during the first 5 d post-weaning. Besides, the distal jejunal protein expression for these two central components in the mitotic and apoptotic response were decreased on d 5 post-weaning. Here, an increased protein expression was rather expected [22, 59]. Nevertheless, based on the major histo-morphological adaptations, it is plausible that weaning resulted in substantial alterations of the epithelial cell turnover [10]. Connected to this, recent advances in redox biology suggest that important cell cycle signaling pathways are under regulation of redox active thiols such as GSH. These mechanisms have been comprehensively reviewed by Circu and Aw [17, 54] and Jones [16], and for example define how a cellular GSH/GSSG E_h_ of over − 220 mV is believed to inhibit cell proliferation. Values of over − 180 mV can activate pro-apoptotic signaling, while values over − 150 mV can result in necrosis. In the current study, where the GSH/GSSG E_h_ was determined in simple mucosal scrapings, this association was not found *in vivo* during the weaning transition. In 3w and 4w weaned piglets for example, the mucosal GSH redox status was found to remain at its steady state level of approximately −240 mV, although massive changes in histo-morphology occurred during the course. Even more, although 3d3w weaned piglets demonstrated a GSH redox imbalance at d 5 post-weaning, amounting to approximately − 220 mV in both the duodenal and distal jejunal mucosa, no critical differences in villus and crypt architecture were observed compared to other weaning treatments at that time point. This aligns with an important study of Tian et al. [60], where *D*,*L*-buthionine-sulfoximine as a specific inhibitor of GSH synthesis was applied in the ileum of rats. This technique inflicted severe mucosal GSH redox imbalance, without provoking changes in histo-morphological indices. Still, we did observe a somewhat dissimilar histo-morphological structure of the small intestine in 3d3w weaned piglets in the current study, but these differences were found to occur already at d 0 and d 2 post-weaning. They are rather believed to result from adaptation to pre-weaning conditions. Deeper crypts and wider villi were for instance found in the pre-weaning period by Vergauwen et al. [31] and De Vos et al. [35].

### Abrupt weaning was associated with barrier disruption, and this co-occurred with redox imbalance

In general, it can be stated that barrier disruption occurred in the small intestine upon weaning in this experiment. Paracellular permeability, assessed by the FD4 flux (4 kDa), peaked on d 2 to d 5 post-weaning in piglets that were artificially reared up till weaning. This substantiates our earlier findings where both transient and long-lasting effects, respectively in the duodenum and the distal jejunum, were observed on the FD4 flux after maternal separation [31]. It should however be noted that the composition of the milk replacer could have impacted on the effect of artificial rearing. For example, Boudry et al. [61] showed that both the protein content and polyunsaturated fatty acids levels [62] could potentially determine the outcome on barrier function. In the current study, the composition of the milk replacer was identical to our previous study [31], and resembles a protein level (249 g/kg crude protein or 50 g/L milk) similar to the low protein diet in Boudry et al. [61]. Vegetable oils in the current milk replacer formula originated from coconut and accordingly are low in polyunsaturated fatty acids. Furthermore, the duodenal FD4 flux was also affected at d 2 post-weaning in piglets that were conventionally raised until 3w of age. Boudry et al. [55] and Hu et al. [63] made similar observations at more distal intestinal sites, in piglets that were weaned at 21 d of age. In contrast, studies that weaned piglets at four to even 7 weeks of age rather report unaltered FD4 fluxes at the mid-small intestine [6, 8]. Indeed, significant work of Smith et al. [7] revealed how weaning age, through stress-induced mast cell degranulation, can impact on the mucosal barrier in the intestine. Altogether, this evidence further confirms that, as observed in this study, weaning age can be an important trigger in post-weaning barrier disruption. To further support our results, the tight junction protein expression of occludin and claudin-3 were assessed in mucosal samples until d 5 post-weaning. These two transmembrane proteins, enabling cell-cell adhesions at the apical side of the intestinal epithelium, play an important role in the regulation of the transcellular barrier [64]. Occludins primary regulate transmembrane transport of uncharged molecules, while claudins control paracellular ion transport [65]. From the claudin family, claudin-3 was analyzed because it also influences paracellular transport of uncharged solutes like FD4 [66]. Bearing in mind the large difference in paracellular permeability between different weaning treatments and the fact that their protein abundance was affected in the pre-weaning period [31], is was hypothesized that these measurements would elaborate more on the functionality of the mucosal barrier [9, 63]. This was however not the case, as average distal jejunal occludin levels were highest in 3d3w weaned piglets and were not affected in the duodenal mucosa. Although weaning treatment did not influence the claudin-3 levels at given day post-weaning, average claudin-3 protein expression was highest in 3d3w weaned piglets. This is similar to or previous study [31], were small intestinal claudin-3 levels were twofold higher in artificially reared versus sow reared piglets at weaning age, although claudin-3 levels were decrease in cooccurrence with barrier dysfunction at maternal separation. Importantly, the FD4 flux only yields the permeability of the whole tissue and not that of its individual components [67], and its interpretations is further complicated by variations in junctional density and the absorptive surface area [68]. Second, not only the absolute tight junction protein quantity but also its phosphorylation status and consequently its distribution can be responsible for the treatment response on FD4 flux [12, 64]. Although this was not assessed in the current study, some observations suggest that this could be the case in the current study, particularly for 3d3w weaned piglets. These animals demonstrated lower GSH stores and a more oxidized GSH redox status in the small intestine throughout the experiment, and particularly demonstrated redox imbalance on d 5 post-weaning. Importantly, oxidative stress is known to affect barrier function through several mechanisms [13, 15], for example by oxidization of redox sensitive protein tyrosine phosphatases (PTPs), which in turn induce phosphorylation and cellular redistribution of tight junction complexes [12]. This would thus imply that redox disturbance and barrier dysfunction have a common cause, being reactive oxygen species that directly induce oxidation of GSH and tight junction regulation proteins. Secondly, protein glutathionylation pathways can directly link the GSH redox system to tight junction functionality. Glutathionylation of specific cysteine residues in mitogen-activated protein kinases (MAPK) [69–71] and PTPs [12, 27, 72] affect their transduction and regulatory properties and ultimately lead to an altered tight junction functionality. Both GSH depletion and GSSG accumulation seem to be crucial events in this mechanism [13, 73]. Here, redox changes in target proteins, initiated by reactive oxygen species or GSH redox imbalance, often precede protein glutathionylation and affects the binding strength of GSH to a protein [74, 75]. Although a close cause-and-effect relationship between redox imbalance and barrier disruption cannot be deduced from the current study, Lou et al. [22] demonstrates that weaning altered MAPK phosphorylation patterns in the liver of piglets, and this cooccurred with oxidative stress. The GSH redox status was however not quantified in that study. Finally, the permeability and size selectivity of the paracellular route in the small intestine potentially also depends on the mucosal surface area accessible to the marker probe. The transepithelial flux of uncharged solutes through crypt tight junctions is far more permeable than in the villous [76–78]. Crypt hyperplasia is therefore likely to contribute to the paracellular the FD4 flux [79]. In the current study, we observed weaning-induced crypt hyperplasia for all weaning treatments. Artificial rearing did not affect crypt depth in the distal jejunum as compared to other weaning treatments, although FD4 permeability was most affected at this site by the 3d3w weaning treatment. Hence, we assume that other factor should account for the observations on paracellular permeability.

The transepithelial HRP flux was employed as measure for transcellular permeability, as intact proteins like HRP are assumed to cross the epithelium mainly via transcytosis [3, 80–84]. Results show that the HRP permeability was not increased in the course of the first 5 d post-weaning. This correlates to research of Spreeuwenberg et al. [8], where also paracellular but not transcellular permeability in the mid-small intestine was increased upon weaning. Indeed, the current study even shows a decrease in HRP permeability when approaching the end of the weaning transition. This was only significantly observed in the duodenum at d 12 and d 28 post weaning, but was already earlier observed by Boudry et al. [55] in the jejunal and ileal mucosa. Most remarkably, post-weaning HRP fluxes were increased in piglets that were artificially reared until weaning at 3w of age. This could correlate to the transient increase in HRP flux that we previously observed shortly after maternal separation [31]. This long-lasting modification of barrier function, most clearly observed in the distal jejunum, draws attention to a risk for more antigen uptake across the intestinal epithelium in these animals. A decrease in HRP flux, normally observed upon weaning [4, 55] and partially confirmed in the current study, is considered to represent an enhancement of the gut maturation process. This is suggested to protect the animal from excessive antigen uptake and activation of the immune system [3]. In important note however is that, in case of severe mucosal damage, HRP can also undergo paracellular transport due to complete loss of tight junction [79, 82, 83, 85]. As long lasting increases of both FD4 and HRP fluxes were observed in the distal jejunum of artificially reared piglets, this observation could signal unrestricted paracellular transport and thus a complete loss of tight junction functionality [79].

## Conclusions

Collectively, these data show that weaning is followed by an upregulation of the GPx and GST activity in the small intestine. Piglets that undergo conventional weaning at either three or four weeks of age manage to maintain GSH redox homeostasis in the small intestinal throughout the weaning transition. In contrast, abrupt transfer from *ad libitum* milk replacer to a cereal-based weaner diet, does result in lower GSH stores throughout the weaning transition, and a GSH redox imbalance on d 5 post-weaning. In these piglets, this co-occurred with an overall defect in the barrier function, which was at its poorest at d 2 to d 5 post-weaning. The fact that massive changes in histo-morphology upon weaning did occur, whereas GSH redox homeostasis was maintained, suggests that the mucosal glutathione redox status does not correlate with small intestinal histo-morphology during the weaning transition.

## Supplementary information

**Additional file 1 Table S1** Composition of the milk replacer and weaner diet, respectively used from 3 d of age until weaning and from d 0 to d 28 post-weaning.

**Additional file 2 Table S2** Overall effects of birht weight (BW), weaning treatment (WT) and days post-weaning (DPW) (*n* = 36-90). **Table S3** The interaction effect of birht weight (BiW) and days post-weaning (DPW) (*n* = 18). **Table S4** The interaction effect of weaning treatment (WT) and days post-weaning (DPW) (*n* = 12). **Table S5** The interaction effect of birht weight (BiW) and weaning treatment (WT) (*n* = 30).

## Data Availability

All analyzed data from this study are included in this published article and its supplementary information files.

## Abbreviations

3d3w: Separated from the sow at 3 d of age and fed a milk replacer until weaning at 3 weeks of age
3w: Three weeks
4w: Four weeks
A: Exposed tissue surface area
ADFI: Average daily feed intake
ADG: Average daily gain
BCA: Bicinchoninic acid assay
Biw: Birth weight
C_0_: Initial concentration
dc/dt: Change in concentration over time
DPW: Days post-weaning
FCR: Feed conversion ratio
FD4: Fluorescein isothiocyanate dextran
GPx: Glutathione peroxidase
GSH: Glutathione
GSH/GSSG E_h_: Glutathione redox status
GSSG: Glutathione disulphide
GST: Glutathione-S-transferase
HRP: Horseradish peroxidase
IUGR: Intrauterine growth regarded
LBW: Low birth weight
MAPK: Mitogen-activated protein kinases
MDA: Malondialdehyde
NBW: Normal birth weight
ORAC: Oxygen radical scavenging ability
P_app_: Apparent permeability coefficient
PCNA: Proliferating cell nuclear antigen
TPTs: Protein tyrosine phosphatases
V: volume
WT: Weaning treatment
