# Supplementary material for: Changes of the glutathione redox system during the weaning transition in piglets, in relation to small intestinal morphology and barrier function
Source: J Anim Sci Biotechnol. 2020 Apr 23;11:45. doi: 10.1186/s40104-020-00440-7 (PMC7178753; doi:10.1186/s40104-020-00440-7)
Supplement: Supplementary file 1 — Additional file 1 Table S1 Composition of the milk replacer and weaner diet, respectively used from 3 d of age until weaning and from d 0 to d 28 post-weaning. [file 40104_2020_440_MOESM1_ESM.docx]

**Table S1** Composition of the milk replacer and weaner diet, respectively used from 3 d of age until weaning and from d 0 to d 28 post-weaning.

|  | **Milk replacer** | **Weaner diet** |
| --- | --- | --- |
| **Ingredient composition, %** |  |  |
| Coco fat filled whey | 42.00 |  |
| Skimmed milk powder | 17.61 |  |
| Whey permeate | 8.29 |  |
| Soy protein concentrate | 10.00 |  |
| Cheddar whey powder | 8.29 |  |
| Whey protein concentrate | 7.00 |  |
| Spray dried blood plasma | 4.00 |  |
| Dicalciumphosphate | 0.32 |  |
| Citric acid | 0.30 |  |
| Barley |  | 33.00 |
| Wheat |  | 19.87 |
| Toasted soybeans |  | 12.00 |
| Soybean meal (49% crude protein) |  | 9.00 |
| Corn |  | 5.00 |
| Acid casein whey powder |  | 4.69 |
| Wheat gluten feed |  | 3.83 |
| Soybean oil |  | 2.22 |
| Lactose |  | 2.00 |
| Sugar beet pulp |  | 2.00 |
| Potato protein |  | 1.71 |
| Lard |  | 1.00 |
| Limestone |  | 0.68 |
| Monocalcium phosphate |  | 0.64 |
| Lactic acid |  | 0.60 |
| *L*-Lysine HCl |  | 0.47 |
| Sodium formiate |  | 0.23 |
| DL-Methionine | 0.31 | 0.21 |
| Calcium formiate |  | 0.20 |
| *L*-Threonine |  | 0.15 |
| *L*-Valine |  | 0.05 |
| *L*-Tryptophane | 0.08 | 0.05 |
| Vitamin and mineral premix | 1.8^1^ | 0.40^2^ |
|  |  |  |
| **Calculated nutrient levels** |  |  |
| Net energy value for pigs, MJ/kg | 15.48 | 9.8 |
| Crude protein, g/kg | 249 | 173 |
| Ether extract, g/kg | 205 | 73 |
| Digestible lysine, g/kg | 18.2 | 11 |
| Digestible methionine + cysteine, g/kg | 11.5 | 6.6 |
| Digestible threonine, g/kg | 11.2 | 6.7 |
| Digestible tryptophane, g/kg | 4.1 | 2.2 |

^1^ The premix supplied as the following (per kg diet): vitamin A, 30,000 IU; vitamin D_3_, 5,000 IU; vitamin E, 75 mg ; Fe^2+^, 120 mg Zn^2+^, 35 mg ; Cu^2+^, 135 mg; Mn^2+^, 45 mg; Se^6+^, 350 µg ; I^﹣^, 1 mg, butylhydroxytoluene, 75 mg/kg.

^2^ The premix supplied as the following (per kg diet): vitamin A, 15,000 IU; vitamin D_3_, 2,000 IU; vitamin E, 100 IU; vitamin K_3_, 2,5 mg; vitamin B_1_, 0,8 mg; vitamin B_2_, 5,6 mg; vitamin B_6_, 0,8 mg ; vitamin B_12_, 26 µg; niacine, 38 mg ; folic acid, 0.4 mg; Fe^2+^, 150 mg Zn^2+^, 115 mg; Cu^2+^, 160 mg; Mn^2+^, 34 mg; Se^6+^, 250 µg ; I^-^, 480 µg, buthylhydroxytoluene, 13,6 mg; Propylgalate, 37.7 mg; ethoxyquin, 1,92 mg; phytase (EC3.1.3.26), 540 FTU; xylanase (3.2.1.8), 70 AXC; glucanase (EC3.2.1.6), 100 AGL.
